# Psychosocial, Neuropsychological, Academic, and Social Outcomes in Pediatric Solid Tumor Survivors: An Exploratory Parent-Reported Study

**DOI:** 10.3390/children13070943

**Published:** 2026-07-18

**Authors:** Paolo Grampa, Annarita Adduci, Lucia Contro, Olga Nigro, Veronica Biassoni, Elisabetta Schiavello, Monica Terenziani, Maura Massimino, Francesco Barretta

**Affiliations:** 1 Pediatric Oncology Unit, Fondazione IRCCS Istituto Nazionale dei Tumori, 20133 Milan, Italy; paolo.grampa@istitutotumori.mi.it (P.G.); annarita.adduci@bp.lnf.it (A.A.); lucia.contro@istitutotumori.mi.it (L.C.); olga.nigro@istitutotumori.mi.it (O.N.); veronica.biassoni@istitutotumori.mi.it (V.B.); elisabetta.schiavello@istitutotumori.mi.it (E.S.); monica.terenziani@istitutotumori.mi.it (M.T.); 2 Department of Biostatistics for Clinical Research, Fondazione IRCCS Istituto Nazionale dei Tumori, 20133 Milan, Italy; francesco.barretta@istitutotumori.mi.it

**Keywords:** socio-economic determinants, pediatric cancer survivors, CNS tumors

## Abstract

**Highlights:**

**What are the main findings?**

- Parent-reported psychological, neuropsychological, social, and academic difficulties were frequent among pediatric cancer survivors when compared with retrospectively reported pre-diagnosis functioning.
- CNS tumor survivors differed from non-CNS survivors in treatment patterns, functional consequences, support needs, social integration difficulties, and cognitive assessment use after cancer.
- In the exploratory screen, no family-related, socio-demographic, clinical, premorbid, or place-based candidate variable remained statistically significantly associated with the eight parent-reported outcomes after false discovery rate correction.

**What are the implications of the main findings?**

- Parent/caregiver-reported functioning and support needs may complement medical surveillance within existing multidisciplinary survivorship follow-up.
- Exploratory patterns involving family educational resources, developmental stage, and premorbid functioning require confirmation in prospective studies using validated multi-informant measures.

**Abstract:**

Background/Objectives: Psychosocial, neuropsychological, social, and academic difficulties may persist after pediatric cancer treatment. We described parent/caregiver-reported functioning and support needs and explored their associations with clinical, family-related, socio-economic, premorbid, and place-based characteristics in an Italian survivorship setting. Methods: This single-center cross-sectional exploratory study included 93 of 130 families approached between November 2022 and January 2023 (response rate, 71.5%). One parent or caregiver completed a purpose-built questionnaire for each survivor. The cohort included 38 survivors with central nervous system (CNS) tumors and 55 with non-CNS tumors. Outcomes were evaluated relative to retrospectively reported pre-diagnosis functioning. Exact confidence intervals, effect estimates, multivariable Firth logistic regression, and Benjamini–Hochberg false discovery rate correction were used. Results: Worsening internalizing difficulties were reported for 48/90 survivors (53.3%), neuropsychological difficulties for 42/90 (46.7%), academic worsening for 30/85 (35.3%), and social integration difficulties for 27/90 (30.0%). CNS survivors more frequently had social integration difficulties than non-CNS survivors (47.4% versus 17.3%; odds ratio, 4.22; 95% confidence interval, 1.50–12.73; q = 0.015) and underwent cognitive assessment after cancer (50.0% versus 17.0%; odds ratio, 4.80; 95% confidence interval, 1.71–14.44; q = 0.013). Municipality size and geographic area showed no nominal associations with parent-reported outcomes. No candidate-variable association in the exploratory screen remained significant after false discovery rate correction. Conclusions: Parent-reported difficulties and support needs were common, with differences by CNS versus non-CNS tumor site. Family-related, premorbid, and place-based patterns are hypothesis-generating and require prospective evaluation using validated multi-informant measures.

## 1. Introduction

Advances in pediatric oncology have led to a substantial increase in long-term survival, shifting clinical and research attention toward the late effects of cancer and its treatment. Among these, psychosocial, neuropsychological, academic, and social difficulties are increasingly recognized as major determinants of long-term functioning and quality of life in childhood cancer survivors [1,2,3,4,5,6]. These difficulties may persist into adolescence and adulthood, influencing educational attainment, interpersonal relationships, and social participation, sometimes to a degree comparable to or exceeding that of physical sequelae [7,8,9,10,11,12]. A large body of literature has documented the role of medical factors—such as tumor location, surgical excision, treatment intensity, exposure to radiotherapy, and neurological complications—in shaping neurocognitive and psychosocial outcomes [5,11,13,14,15,16]. Survivors of central nervous system (CNS) tumors, in particular, have consistently been shown to be at increased risk for cognitive impairment, emotional difficulties, academic decline, and social maladjustment. In parallel, individual and family-level variables, including parent–child communication, attachment quality, illness awareness, and coping strategies, have been identified as important protective or risk-modifying factors [6,17,18,19,20,21,22,23,24,25,26,27].

Although clinical and family-related predictors have been widely investigated, considerably less attention has been devoted to broader Social Determinants of Health (SDOH) that may shape survivorship outcomes beyond disease-related characteristics [28]. Contemporary SDOH frameworks recognize that health outcomes are shaped not only by biological and clinical factors but also by the social, economic, and environmental conditions in which children live, grow, and receive care [29]. This perspective is increasingly reflected in pediatric health research, where SDOH are recognized as important contributors to developmental, educational, and psychosocial outcomes independently of medical factors [30,31].

Accordingly, survivorship outcomes may reflect not only treatment-related late effects but also differences in the social and environmental contexts that influence children’s opportunities to receive rehabilitation, educational accommodations, neuropsychological follow-up, and community support [28,32,33]. Within pediatric oncology survivorship, these determinants may influence access to rehabilitation services, neuropsychological assessment, educational support, and opportunities for social participation, thereby affecting long-term functional outcomes beyond disease-related characteristics. In the general pediatric population and among children with acquired brain injuries, socioeconomic conditions—particularly parental education—are robustly associated with cognitive development, emotional regulation, and adaptive functioning [34,35]. However, their role in pediatric cancer survivorship remains less clearly defined, especially when family educational background, cultural context, and broader contextual characteristics are considered alongside traditional economic indicators such as income or occupation [5,11,16,27,28,36,37,38,39].

Validated multidimensional approaches, such as the Psychosocial Assessment Tool, illustrate how family psychosocial risk may be assessed across domains including family structure, social support, caregiver stress, and child or sibling difficulties [40]. Health literacy and the ability to understand and navigate complex care pathways represent additional dimensions of family resources that were not directly measured in the present study.

Among contextual determinants, place-based characteristics deserve particular attention because they capture aspects of the environments in which children live and receive healthcare that are not fully reflected by individual socioeconomic indicators. Geographic area of residence and municipality size have been used as contextual proxy indicators of place-based SDOH in population health and epidemiological research, particularly when more granular neighborhood-level measures are unavailable [28,30,34]. Although these variables are not direct measures of individual socioeconomic status or neighborhood deprivation, they may capture contextual differences in healthcare accessibility, educational resources, transportation networks, availability of specialized rehabilitation and neuropsychological services, and opportunities for social participation [28,34].

This issue is particularly relevant in Italy, where universal healthcare coverage is guaranteed by the National Health Service (Servizio Sanitario Nazionale), but responsibility for healthcare organization is largely devolved to regional authorities. This regional organization may contribute to contextual differences in the practical accessibility of specialist services, particularly outside larger urban centers, although these factors were not measured in this study. In the present study, municipality size was considered a pragmatic proxy for degree of urbanicity and local service context, rather than a direct measure of proximity to referral centers or access to multidisciplinary survivorship care [28]. This distinction is relevant even within a universal healthcare system, where formal entitlement to care does not necessarily ensure comparable local availability or practical accessibility of specialist services [41]. Neither municipality size nor geographic macro-area captures actual travel burden, local service availability, neighborhood deprivation, or individual use of healthcare and educational resources; therefore, these variables were interpreted as broad contextual indicators only.

Despite growing interest in SDOH, evidence regarding the contribution of these contextual characteristics to pediatric cancer survivorship remains limited, particularly within Southern European healthcare systems [28]. Most available studies originate from North America or Northern Europe, where healthcare organization and social welfare systems differ substantially from the Italian context. Within this conceptual framework, the present study explored whether selected family-level and contextual characteristics, including proxy indicators of place-based SDOH, were associated with parent-reported psychological, neuropsychological, academic, and social outcomes among survivors of pediatric cancers, including CNS tumors. The objectives were twofold: (i) to describe psychological, neuropsychological, academic, and social outcomes after completion of therapy, and (ii) to explore their associations with family, cultural, socioeconomic, and place-based characteristics, as well as premorbid vulnerabilities. We hypothesized that family educational level and pre-existing psychological or scholastic difficulties might show stronger exploratory associations with post-treatment functioning than broader contextual proxy indicators such as geographic residence and municipality size.

## 2. Methods

### 2.1. Study Design and Participants

This was a single-center cross-sectional exploratory study. In the study period (November 2022–January 2023), an anonymous, purpose-built 60-item questionnaire was administered to parents of survivors who had completed therapy and were attending routine follow-up at our pediatric outpatient clinic (Supplementary Material File S1).

The survivor was the unit of analysis. One questionnaire was collected for each survivor and completed by one parent/caregiver, either individually or jointly with another caregiver.

### 2.2. Questionnaire and Data Collection

A purpose-built 60-item parent/caregiver questionnaire was used to collect information on survivor functioning, post-treatment support needs, family context, socio-economic circumstances, educational background, and selected premorbid characteristics (Supplementary Material File S1).

The questionnaire was developed for exploratory data collection in the local clinical setting and was not intended as a validated patient-reported outcome measure, diagnostic instrument, or substitute for standardized psychological or neuropsychological assessment. Most items were factual, checklist-based, dichotomous, categorical, or ordinal indicators. Questionnaire-derived measures were therefore interpreted as parent/caregiver-reported contextual indicators rather than clinical diagnoses or standardized severity measures.

The questionnaire included items referring to current or post-treatment functioning and, for selected constructs, retrospectively reported pre-diagnosis functioning. Parents/caregivers completed the questionnaire on the basis of their knowledge of the survivor’s everyday functioning and family circumstances.

### 2.3. Questionnaire-Derived Outcomes and Candidate Variables

The questionnaire addressed four parent-reported functional domains: psychological, neuropsychological, social, and academic functioning. It also collected information on post-treatment psychological and cognitive assessment use. Operational definitions of the parent-reported outcomes are provided in Supplementary Table S1.

Candidate variables were organized into demographic, clinical, family-related, cultural, socio-economic, premorbid, and place-based contextual domains. Geographic area of residence and municipality size were considered broad contextual proxies rather than direct measures of neighborhood deprivation, travel burden, local service availability, or healthcare utilization. Detailed coding rules, permissible ranges, and the analytical role of each questionnaire-derived variable are reported in Supplementary Material File S2 and Supplementary Table S2.

### 2.4. Statistical Analysis

Analyses were based on available data, without imputation of missing values. Item-level completeness of the questionnaire response fields is reported in Supplementary Table S3. Categorical variables are summarized as frequencies and percentages, and continuous variables as median and first and third quartiles (Q1–Q3). For binary analyses, parent-reported functional outcomes were coded as worsening versus stable or improved functioning, based on the comparison between parent-reported post-treatment/current status and retrospectively reported pre-diagnosis functioning. Psychological and cognitive assessments after cancer were analyzed separately as indicators of reported assessment use and were not treated as functional outcomes.

Prevalences of parent-reported functional outcomes and post-treatment assessment use were estimated with exact 95% binomial confidence intervals using the Clopper–Pearson method [42].

The comparison between survivors of CNS and non-CNS tumors was specified a priori, based on the expected differential burden of neurocognitive and psychosocial late effects among CNS tumor survivors. This comparison was applied to treatment patterns, questionnaire-derived functional consequences and support needs, parent-reported functional outcomes, and post-treatment assessment use. For categorical variables with more than two levels, distributions were compared using Fisher’s exact test; for r × c tables, Monte Carlo *p*-values were calculated using 20,000 replicates. The strength of association was quantified using Cramér’s V, with 95% confidence intervals obtained by nonparametric bootstrap resampling with 2000 replicates [43].

For binary outcomes, CNS and non-CNS groups were compared using Fisher’s exact test. Absolute differences in prevalence were expressed as risk differences, calculated as the CNS minus non-CNS prevalence, with 95% confidence intervals obtained using the Newcombe–Wilson method [44]. Odds ratios and corresponding exact 95% confidence intervals were derived from Fisher’s exact test.

Because of the limited sample size, sparse outcome data for some comparisons, and the potential for separation, logistic regression analyses were performed using Firth penalized likelihood [45,46]. Odds ratios, profile penalized-likelihood 95% confidence intervals, and two-sided *p*-values are reported.

Three multivariable models were specified for parent-reported worsening of externalizing difficulties, neuropsychological difficulties, and academic worsening. The externalizing-difficulties model included family educational level, premorbid externalizing difficulties, tumor site, sex, and parental separation. The neuropsychological-difficulties model included tumor site, age at diagnosis, multimodal treatment, family educational level, and premorbid neuropsychological difficulties. The academic-worsening model included tumor site, age at diagnosis, multimodal treatment, family educational level, and premorbid PEI/PDP. These models were considered exploratory adjusted association models and were not intended for causal inference or prediction.

An univariable exploratory screen was additionally conducted for 15 selected candidate variables across eight parent-reported outcomes. Binary and continuous candidate variables were analyzed using univariable Firth logistic regression. Municipality size was analyzed as a binary predictor, whereas geographic area of residence was evaluated using a global Monte Carlo Fisher–Freeman–Halton test across Northern, Central, and Southern Italy because of sparse geographic strata. Given the limited sample size, the residential variables were not included in the multivariable models. Results should be interpreted as hypothesis-generating.

To account for multiple testing, *p*-values were adjusted using the Benjamini–Hochberg false discovery rate procedure [47]. Adjustments were performed separately for CNS versus non-CNS comparisons, CNS versus non-CNS outcome comparisons, the coefficients across the three multivariable models, and the associations in the univariable exploratory screen. Nominal *p*-values and FDR-adjusted q-values are reported. All statistical tests were two-sided.

The analyses were conducted using SAS Studio (Release 5.2) and R (Available online: http://www.r-project.org/ (accessed on 6 July 2026), R version 4.5.1).

## 3. Results

Of 130 families approached, 93 completed a questionnaire (response rate, 71.5%). One questionnaire was available for each survivor and was completed by a parent or caregiver. Survivor, respondent, and household characteristics are reported in Table 1. Questionnaires were completed by mothers in 51 cases (54.8%), fathers in 24 (25.8%), and both parents or another caregiver in 18 (19.4%). The survivor cohort comprised 49 males (52.7%) and 44 females (47.3%). At questionnaire completion, the median age was 16.2 years (Q1-Q3, 10.9–20.5). Median time since diagnosis was 52.5 months (Q1-Q3, 27.9–76.8; *n* = 91), and median age at diagnosis was 11.8 years (Q1-Q3, 6.0–15.5; *n* = 92). Most survivors were students (78/93, 83.9%), whereas 9 (9.7%) were employed and 5 (5.4%) were unemployed, and current activity was not specified for 1 survivor (1.1%). Place-based contextual characteristics are reported in Table 1. Municipality size was available for 90 survivors: 69/90 (76.7%) lived in small municipalities and 21/90 (23.3%) in larger municipalities or cities. Geographic area of residence was available for 90 survivors; 77/90 (85.6%) resided in Northern Italy, 2/90 (2.2%) in Central Italy, and 11/90 (12.2%) in Southern Italy.

Thirty-eight survivors (40.9%) had CNS tumors and 55 (59.1%) had non-CNS tumors. Treatment consisted of surgery, chemotherapy, and radiotherapy in 29 cases (31.2%), surgery plus chemotherapy in 19 (20.4%), surgery alone in 24 (25.8%), and other treatment patterns or no treatment in 21 (22.6%). Detailed family structure, parental occupation, and illness-related financial support are reported in Supplementary Table S4, whereas retrospectively reported pre-diagnosis developmental, psychological, social, and educational characteristics are reported in Supplementary Table S5.

Detailed clinical characteristics and post-treatment support needs are reported in Supplementary Table S6. Among 92 survivors with available data, appearance- and growth-related consequences were absent in 43 (46.7%), involved one affected domain in 41 (44.6%), and involved two affected domains in 8 (8.7%). Learning and language-related difficulties were absent in 53/92 survivors (57.6%), involved one affected domain in 26 (28.3%), two domains in 9 (9.8%), three domains in 3 (3.3%), and four domains in 1 (1.1%). Physical rehabilitation needs were reported for 33/92 survivors (35.9%), neurodevelopmental rehabilitation needs for 20/92 (21.7%), and psychological/educational intervention needs for 25/92 (27.2%).

**Figure 1:**
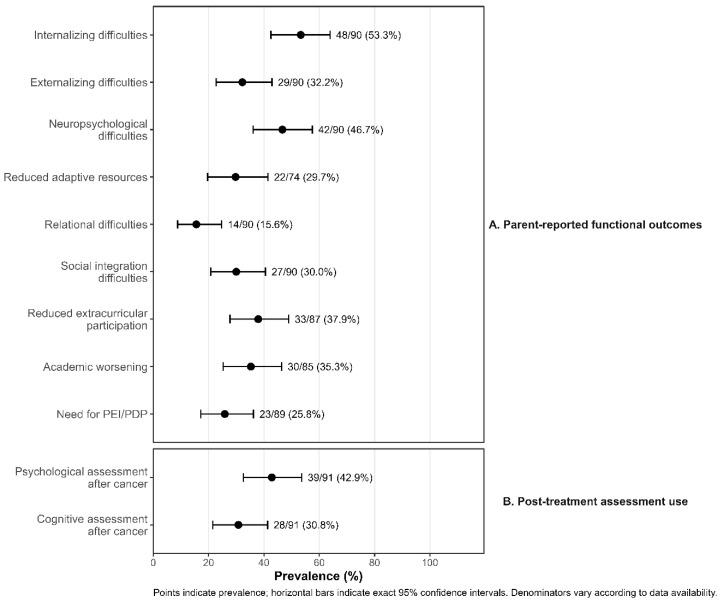
Parent-reported functional outcomes and post-treatment assessment use among pediatric cancer survivors. *Panel A shows the prevalence of parent-reported worsening or new difficulties relative to retrospectively reported pre-diagnosis functioning. Panel B shows the proportion of survivors reported to have undergone psychological or cognitive assessment after cancer. Points indicate prevalence and horizontal bars indicate exact 95% confidence intervals. Denominators vary according to data availability.*

Parent-reported functional outcomes and post-treatment assessment use are summarized in Figure 1, with exact 95% CI. Relative to retrospectively reported pre-diagnosis functioning, worsening of internalizing difficulties was reported for 48/90 survivors (53.3%; 95% CI, 42.5–63.9), externalizing difficulties for 29/90 (32.2%; 95% CI, 22.8–42.9), and neuropsychological difficulties for 42/90 (46.7%; 95% CI, 36.1–57.5). A reduction in adaptive resources was reported for 22/74 survivors (29.7%; 95% CI, 19.7–41.5). Relational difficulties were reported for 14/90 survivors (15.6%; 95% CI, 8.8–24.7), social integration difficulties for 27/90 (30.0%; 95% CI, 20.8–40.6), and a reduction in extracurricular activities for 33/87 (37.9%; 95% CI, 27.7–49.0). Academic worsening was reported for 30/85 survivors (35.3%; 95% CI, 25.2–46.4), while 23/89 (25.8%; 95% CI, 17.1–36.2) had a new or worsened need for an Individualized Education Plan or Personalized Didactic Plan. Psychological assessment after cancer was reported for 39/91 survivors (42.9%; 95% CI, 32.5–53.7), and cognitive assessment after cancer for 28/91 (30.8%; 95% CI, 21.5–41.3).

Comparisons between CNS and non-CNS survivors are reported in Table 2 and Table 3. The distribution of oncological treatment differed between groups (Cramér’s V = 0.38, 95% CI, 0.27–0.55; q = 0.005), with surgery, chemotherapy, and radiotherapy used in 16/38 CNS survivors (42.1%) and 13/55 non-CNS survivors (23.6%). CNS survivors also differed from non-CNS survivors in the distribution of appearance- and growth-related consequences (Cramér’s V = 0.29, 95% CI, 0.13–0.46; q = 0.032), learning and language-related difficulties (Cramér’s V = 0.37, 95% CI, 0.23–0.55; q = 0.013), physical rehabilitation needs (Cramér’s V = 0.28, 95% CI, 0.11–0.49; q = 0.032), neurodevelopmental rehabilitation needs (Cramér’s V = 0.38, 95% CI, 0.25–0.53; q = 0.005), and psychological/educational intervention needs (Cramér’s V = 0.27, 95% CI, 0.11–0.45; q = 0.032).

Among parent-reported outcomes, social integration difficulties were reported more frequently among CNS than non-CNS survivors: 18/38 (47.4%) versus 9/52 (17.3%), corresponding to a risk difference of 30.1 percentage points (95% CI, 2.7–53.4) and an odds ratio of 4.22 (95% CI, 1.50–12.73; q = 0.015). Cognitive assessment after cancer was also more frequently reported among CNS survivors, occurring in 19/38 (50.0%) compared with 9/53 non-CNS survivors (17.0%) (risk difference, 33.0 percentage points; 95% CI, 5.6–56.0; OR, 4.80; 95% CI, 1.71–14.44; q = 0.013). Other outcome differences did not remain statistically significant after Benjamini–Hochberg correction.

Three multivariable Firth logistic regression models were fitted for externalizing difficulties, neuropsychological difficulties, and academic worsening (Table 4). No association remained statistically significant after correction for multiple testing across the multivariable models. Nevertheless, several estimates showed nominal associations that may be relevant for future investigation. Middle/high family educational level was associated with lower estimated odds of parent-reported externalizing difficulties (adjusted OR, 0.44; 95% CI, 0.16–1.12; *p* = 0.086; q = 0.214) and academic worsening (adjusted OR, 0.41; 95% CI, 0.14–1.11; *p* = 0.080; q = 0.214). Older age at diagnosis was associated with lower odds of parent-reported neuropsychological difficulties (adjusted OR per interquartile-range increase, 0.49; 95% CI, 0.24–0.94; *p* = 0.032; q = 0.214) and academic worsening (adjusted OR per interquartile-range increase, 0.27; 95% CI, 0.10–0.66; *p* = 0.003; q = 0.052). These estimates were based on 89 complete cases for the externalizing and neuropsychological models and 83 complete cases for the academic-worsening model.

The complete exploratory univariable screen is reported in Supplementary Table S7. None of the 120 screened candidate variable-outcome associations remained statistically significant after Benjamini–Hochberg correction. In particular, municipality size and geographic area of residence did not show nominal associations with parent-reported psychological, neuropsychological, academic, or social outcomes in the exploratory univariable screen. Estimates for geographic area should be interpreted cautiously because only 13 survivors resided outside Northern Italy, including only two in Central Italy. By contrast, a greater number of siblings was nominally associated with higher odds of parent-reported neuropsychological difficulties (OR per interquartile-range increase, 1.77; 95% CI, 1.07–3.19; *p* = 0.027; q = 0.394), while premorbid internalizing difficulties were nominally associated with a new or worsened need for an Individualized Education Plan or Personalized Didactic Plan after cancer (OR, 3.15; 95% CI, 1.20–8.64; *p* = 0.020; q = 0.344).

## 4. Discussion

The present study explored psychosocial, neuropsychological, and academic outcomes in a cohort of pediatric cancer survivors, using a purpose-built parent/caregiver questionnaire and retrospective comparison with pre-diagnosis functioning. Comparisons between CNS and non-CNS tumor survivors showed differences in treatment patterns, appearance- and growth-related consequences, learning and language-related difficulties, and rehabilitation or support needs, consistent with previous literature describing the neurocognitive and psychosocial vulnerability of CNS tumor survivors [5,11,13,14,15,16]. Direct numerical comparison with prior cohorts is limited by the non-validated parent/caregiver questionnaire and retrospective pre-diagnosis reference used in this study. Nevertheless, the observed frequencies of internalizing, neuropsychological, academic, and social difficulties are broadly consistent with the recognized burden of emotional, cognitive, educational, and social late effects described in pediatric cancer survivorship studies [48,49]. Several nominal associations with family, socio-demographic, clinical, and premorbid characteristics emerged in the exploratory analyses; however, none remained statistically significant after false discovery rate correction. These associations should therefore be considered hypothesis-generating rather than evidence of independent variables of post-treatment functioning. Although no family-related, socio-demographic, or premorbid characteristic remained statistically significant after false discovery rate adjustment, several exploratory estimates were directionally consistent with the possibility that post-treatment functioning is influenced by factors beyond tumor-related characteristics alone. Middle/high family educational level was associated with lower estimated odds of parent-reported externalizing difficulties and academic worsening in the multivariable adjusted models, although these estimates were imprecise and did not meet the adjusted significance threshold. The absence of an FDR-significant association for family educational level should not be interpreted as evidence that socio-economic resources are irrelevant; prior survivorship studies have linked family-and neighborhood-level social determinants with patient-reported outcomes, cognitive outcomes, and school performance [49,50,51]. Rather, this null finding should be interpreted in light of the small sample size, limited power, composite nature of the education variable, absence of direct health-literacy or deprivation measures, and multiple-testing correction. In the univariable exploratory screen, a greater number of siblings was associated with higher odds of parent-reported neuropsychological difficulties, while premorbid internalizing difficulties were associated with a new or worsened need for PEI/PDP after cancer. The sibling-number estimate may also reflect unmeasured family, cultural, or reporting-related factors rather than a direct effect of family size. Older age at diagnosis was also associated with lower estimated odds of neuropsychological difficulties and academic worsening in the focused models. These findings should not be interpreted as established independent risk or protective factors; rather, they identify potentially relevant developmental, family, and educational pathways that warrant prospective investigation using validated measures in larger cohorts.

Null residential findings do not establish that place-based factors are unimportant: these broad proxies did not capture travel burden or local service availability, neighborhood deprivation, or actual healthcare use, and only 13 survivors lived outside Northern Italy.

Among the parent-reported outcomes, social integration difficulties were more frequent among CNS survivors than among non-CNS survivors, and cognitive assessment after cancer was also more frequently reported in the CNS group. Assessment use and parent-reported difficulties capture different constructs; consequently, the observed prevalence of psychological or cognitive assessment after cancer cannot be interpreted as a direct measure of the adequacy of follow-up care. Several limitations must be acknowledged. The questionnaire used in this study was purpose-built and not formally validated, and outcomes were based on parental report rather than standardized assessments. The psychometric properties of the questionnaire, including construct validity, convergent and discriminant validity, test–retest reliability, responsiveness, and measurement error, were not evaluated; therefore, prevalence estimates should be interpreted as descriptive parent/caregiver-reported indicators rather than estimates of clinically defined disorders. The cross-sectional design limits causal inference and relies on retrospective comparison with pre-diagnosis functioning, which may be subject to recall bias. Although multivariable Firth logistic models were fitted, the limited sample size and number of complete cases constrained model complexity and the precision of the estimates. Firth regression reduces small-sample bias but cannot compensate for limited information, and these models should not be considered to provide comprehensive confounding control. Potentially relevant variables, including time since diagnosis, respondent type, additional socio-economic indicators, rehabilitation exposure, age at questionnaire, and more detailed treatment exposures, could not be incorporated without overfitting and further instability. Family educational level was a composite measure based on available parental and grandparental education and could not distinguish the contribution of individual caregivers or other dimensions of socioeconomic resources. Treatment was represented by a composite multimodal-treatment indicator rather than treatment-specific exposures; residual confounding by radiotherapy and other clinical factors cannot therefore be excluded. The exploratory analyses involved multiple comparisons, and no association remained statistically significant after false discovery rate correction. This absence of FDR-significant findings should also be interpreted as evidence of limited power for broad hypothesis testing. Finally, 93 of 130 contacted families completed the questionnaire; participation bias cannot be excluded, and the single-center design limits generalizability.

## 5. Conclusions

This study documents frequent parent-reported psychological, neuropsychological, social, and academic difficulties among pediatric cancer survivors, together with differences in selected clinical characteristics, support needs, social integration difficulties, and cognitive assessment use according to CNS versus non-CNS tumor site. Although no family-related, socio-demographic, clinical, premorbid, or place-based characteristic variable-outcome association in the exploratory screen remained statistically significant after adjustment for multiple testing, the exploratory patterns observed suggest that developmental stage, family educational resources, premorbid functioning, and broader family context may merit further consideration in prospective survivorship research. Future research should employ validated instruments, longitudinal designs, and multicenter samples to clarify causal pathways and to determine whether the observed patterns extend across different healthcare and cultural contexts. In conclusion, pediatric cancer survivorship care should combine medical surveillance with attention to parent-reported functioning and the broader family context, while avoiding the interpretation of specific family characteristics as established risk factors on the basis of the present exploratory data.

## Figures and Tables

**Table 1:** Characteristics of survivors, parent/caregiver respondents, and households.

Section	Characteristic	Overall, *n* (%)
		N = 93
**Survivor characteristics**		
	**Sex**	
	Female	44 (47.3)
	Male	49 (52.7)
	**Age at questionnaire completion, years**	
	Median (first and third quartiles)	16.2 (10.9–20.5)
	**Current main activity**	
	Employed	9 (9.7)
	Student	78 (83.9)
	Unemployed	5 (5.4)
	Not specified	1 (1.1)
**Clinical characteristics**		
	**Tumor site**	
	CNS	38 (40.9)
	Non-CNS tumors	55 (59.1)
	Age at diagnosis, years *	
	Median (first and third quartiles)	11.8 (6.0–15.5)
	Time since diagnosis, months **	
	Median (first and third quartiles)	52.5 (27.9–76.8)
	**Treatment**	
	Other treatment or none	21 (22.6)
	Surgery only	24 (25.8)
	Surgery plus chemotherapy	19 (20.4)
	Surgery, chemotherapy, and radiotherapy	29 (31.2)
**Respondent and household characteristics**		
	**Questionnaire respondent**	
	Father	24 (25.8)
	Mother	51 (54.8)
	Both parents/other caregiver	18 (19.4)
	**Parents’ marital status**	
	Cohabiting	77 (82.8)
	Separated	11 (11.8)
	Other	5 (5.4)
	Maternal age, years *	
	Median (first and third quartiles)	49.5 (44.0–55.0)
	Paternal age, years ***	
	Median (first and third quartiles)	51.0 (46.0–56.8)
	Family educational level *	
	Mid/High	39 (42.4)
	Mid/Low	53 (57.6)
	**Household income sources before illness**	
	High	10 (10.8)
	Low	20 (21.5)
	Middle	63 (67.7)
	**Any siblings**	
	No	27 (29.0)
	Yes	66 (71.0)
	**Municipality size**	
	Larger municipality/city (>20,000 inhabitants)	21 (22.6)
	Small municipality (≤20,000 inhabitants)	69 (74.2)
	Not specified	3 (3.2)
	**Geographic residence**	
	Northern Italy	77 (82.8)
	Central Italy	2 (2.2)
	Southern Italy	11 (11.8)
	Not specified	3 (3.2)

*Abbreviations:* CNS, central nervous system. * One missing value. ** Two missing values. *** Three missing values.

**Table 2:** Clinical characteristics, functional consequences, and rehabilitation needs according to CNS versus non-CNS tumor status.

Characteristic	CNS, *n* (%)	Non-CNS, *n* (%)	Cramér’s V (95% CI) *	*p*-Value **	FDR-Adjusted q Value ***
**Oncological treatment**			0.382 (0.269–0.546)	0.002	0.005
Other treatment or none	11 (28.9)	10 (18.2)			
Surgery only	10 (26.3)	14 (25.5)			
Surgery plus chemotherapy	1 (2.6)	18 (32.7)			
Surgery, chemotherapy, and radiotherapy	16 (42.1)	13 (23.6)			
Appearance and growth-related consequences ****			0.292 (0.126–0.463)	0.022	0.032
0	15 (39.5)	28 (51.9)			
1	16 (42.1)	25 (46.3)			
2	7 (18.4)	1 (1.9)			
Learning and language-related difficulties ****			0.370 (0.235–0.546)	0.007	0.013
0	15 (39.5)	38 (70.4)			
1	13 (34.2)	13 (24.1)			
2	6 (15.8)	3 (5.6)			
3	3 (7.9)	0 (0.0)			
4	1 (2.6)	0 (0.0)			
Physical rehabilitation needs ****			0.281 (0.110–0.487)	0.031	0.032
0	20 (52.6)	39 (72.2)			
1	15 (39.5)	8 (14.8)			
2	3 (7.9)	7 (13.0)			
Neurodevelopmental rehabilitation needs ****			0.385 (0.255–0.528)	0.001	0.005
0	24 (63.2)	48 (88.9)			
1	6 (15.8)	6 (11.1)			
2	5 (13.2)	0 (0.0)			
3	3 (7.9)	0 (0.0)			
Psychological/educational intervention needs ****			0.270 (0.109–0.451)	0.032	0.032
0	23 (60.5)	44 (81.5)			
1	10 (26.3)	9 (16.7)			
2	5 (13.2)	1 (1.9)			

*Abbreviations:* CNS, central nervous system; CI, confidence interval; FDR, false discovery rate. * Cramér’s V confidence intervals were obtained by bootstrap. ** *p*-values refer to the overall comparison of distributions between CNS and non-CNS survivors. *** Q-values were calculated using the Benjamini–Hochberg procedure across the six comparisons. **** One missing value in non-CNS group. Levels 0–2/3/4 indicate the number of reported affected domains or needs within the relevant construct.

**Table 3:** Parent-reported functional outcomes and post-treatment assessment use according to tumor site.

Outcome	CNS Tumors, *n*/N (%)	Non-CNS Tumors, *n*/N (%)	Risk Difference, Percentage Points (95% CI) *	Odds Ratio (95% CI) **	*p* Value	FDR-Adjusted q Value ***
**Parent-reported functional outcomes**						
Worsening of internalizing problems	22/38 (57.9)	26/52 (50.0)	7.9 (−20.9 to 35.3)	1.37 (0.55–3.48)	0.524	0.622
Worsening of externalizing problems	15/38 (39.5)	14/52 (26.9)	12.6 (−14.7 to 38.5)	1.76 (0.66–4.76)	0.256	0.402
Worsening of neuropsychological problems	22/38 (57.9)	20/52 (38.5)	19.4 (−9.8 to 45.7)	2.18 (0.86–5.64)	0.088	0.191
Reduction in adaptive resources	13/30 (43.3)	9/44 (20.5)	22.9 (−7.1 to 49.6)	2.93 (0.95–9.50)	0.042	0.115
Worsening of relational problems	7/38 (18.4)	7/52 (13.5)	5.0 (−16.1 to 26.7)	1.45 (0.39–5.37)	0.566	0.622
Worsening of social integration difficulties	18/38 (47.4)	9/52 (17.3)	30.1 (2.7–53.4)	4.22 (1.50–12.73)	0.003	0.015
Reduction in extracurricular activities	13/34 (38.2)	20/53 (37.7)	0.5 (−27.3 to 29.0)	1.02 (0.38–2.70)	1.000	1.000
Worsening of academic performance	16/34 (47.1)	14/51 (27.5)	19.6 (−9.5 to 46.1)	2.32 (0.86–6.45)	0.104	0.191
New or worsened need for PEI/PDP	11/35 (31.4)	12/54 (22.2)	9.2 (−16.4 to 34.8)	1.60 (0.55–4.65)	0.458	0.622
**Post-treatment assessment use**						
Psychological assessment after cancer	22/38 (57.9)	17/53 (32.1)	25.8 (−3.3 to 51.1)	2.88 (1.13–7.57)	0.019	0.068
Cognitive assessment after cancer	19/38 (50.0)	9/53 (17.0)	33.0 (5.6–56.0)	4.80 (1.71–14.44)	0.001	0.013

*Abbreviations:* CI, confidence interval; CNS, central nervous system; FDR, false discovery rate; PDP, Personalized Didactic Plan; PEI, Individualized Education Plan. * Risk differences are expressed as CNS minus non-CNS prevalence; positive values indicate a higher prevalence among CNS survivors. ** Odds ratios and *p* values were obtained from Fisher’s exact tests. *** Benjamini–Hochberg adjustment across the 11 comparisons.

**Table 4:** Multivariable Firth logistic regression models for selected parent-reported outcomes.

Outcome and Covariate	Adjusted OR (95% CI) *	*p* Value *	FDR-Adjusted q Value **
**Parent-reported worsening of externalizing problems** *Complete cases: n = 89; events: n = 28*			
**Family education**			
Middle/high vs. Middle/low	0.44 (0.16–1.12)	0.086	0.214
**Premorbid externalizing problems**			
Any vs. None	0.60 (0.20–1.68)	0.340	0.464
**Tumor site**			
CNS tumor vs. Non-CNS tumor	1.58 (0.63–3.98)	0.330	0.464
**Sex**			
Female vs. Male	0.67 (0.26–1.69)	0.402	0.503
**Parental separation**			
Yes vs. No	2.05 (0.54–7.72)	0.281	0.464
**Parent-reported worsening of neuropsychological problems** *Complete cases: n = 89; events: n = 41*			
**Tumor site**			
CNS tumor vs Non-CNS tumor	2.40 (0.98–6.14)	0.056	0.214
**Age at diagnosis**			
15.5 vs. 6.0 ***	0.49 (0.24–0.94)	0.032	0.214
**Treatment**			
Surgery, chemotherapy, and radiotherapy vs. all other treatment patterns	1.32 (0.50–3.45)	0.566	0.653
**Family education**			
Middle/high vs. Middle/low	0.58 (0.24–1.39)	0.222	0.417
**Premorbid neuropsychological problems**			
Any vs. None	0.82 (0.29–2.26)	0.704	0.754
**Parent-reported academic worsening** *Complete cases: n = 83; events: n = 29*			
**Tumor site**			
CNS tumor vs Non-CNS tumor	2.47 (0.90–7.14)	0.081	0.214
**Age at diagnosis**			
15.5 vs. 6.0 ***	0.27 (0.10–0.66)	0.003	0.052
**Treatment**			
Surgery, chemotherapy, and radiotherapy vs. all other treatment patterns	2.25 (0.78–6.49)	0.134	0.287
**Family education**			
Middle/high vs. Middle/low	0.41 (0.14–1.11)	0.080	0.214
**Premorbid PEI/PDP**			
Yes vs. No	1.10 (0.21–4.88)	0.904	0.904

*Abbreviations:* CI, confidence interval; CNS, central nervous system; FDR, false discovery rate; OR, odds ratio; PDP, Personalized Didactic Plan; PEI, Individualized Education Plan. * Adjusted odds ratios, 95% confidence intervals, and *p* values were estimated using Firth penalized logistic regression. ** Benjamini–Hochberg adjustment across all 15 coefficients from the three models. *** Values represent first and third quartile of the variable distribution.

## Data Availability

The original contributions presented in this study are included in the article/Supplementary Material. Further inquiries can be directed to the corresponding author.

## Supplementary Materials

The following supporting information can be downloaded at: https://www.mdpi.com/article/10.3390/children13070943/s1, File S1: “OLTRE” questionnaire (English translation); File S2: Operational definitions and coding rules; Table S1: Operational definitions of parent-reported functional outcomes and post-treatment assessment use; Table S2: Questionnaire-derived candidate variables, operational definitions, and role in the analyses; Table S3: Completeness of raw questionnaire response fields summarized by questionnaire item or item block; Table S4: Detailed family structure, parental occupation, and illness-related financial support not reported in Table 1; Table S5: Retrospectively reported pre-diagnosis developmental, psychological, social, and educational characteristics; Table S6: Detailed clinical profile and post-treatment support needs not reported in Table 1; Table S7: Exploratory univariable analyses of candidate variables and parent-reported outcomes.
