# Supplementary material for: Psychosocial, Neuropsychological, Academic, and Social Outcomes in Pediatric Solid Tumor Survivors: An Exploratory Parent-Reported Study
Source: Children (Basel). 2026 Jul 18;13(7):943. doi: 10.3390/children13070943 (PMC13406780; doi:10.3390/children13070943)
Supplement: Supplementary file 1 [file children-13-00943-s001.zip › Supplementary Material File S1.pdf]

Date of questionnaire completion: ……………………………………………………………………………

Who is completing the questionnaire?

1 ( ) Mother 2 ( ) Father 3 ( ) Both parents 4 ( ) Other: …………………………………

PATIENT INFORMATION

Month and year of birth: …………………………………………………… **Age:** ………………………

Gender: 1 ( ) Male 2 ( ) Female 3 ( ) Other / prefer not to answer

Residence: 1 ( ) North 2 ( ) Center 3 ( ) South

1 ( ) Small town, up to ~20,000 inhabitants 2 ( ) Larger town/city, more than 20,000 inhabitants

**CURRENT STATUS OF THE PATIENT**		
• STUDENT	SCHOOL ATTENDED:<br><br>1 ( ) Kindergarten (year: ………………)<br>2 ( ) Primary school (grade: ………………)<br>3 ( ) Lower secondary school (grade: ………………)<br>4 ( ) Upper secondary school (grade: ………………)<br>5 ( ) University (…………………………)<br>6 ( ) Postgraduate program (second-level Master's, PhD/Doctorate, specialization)	
• EMPLOYED<br>1 ( ) Temporary / Precarious position<br>2 ( ) Permanent position	A ( ) Manual worker / Craftsperson<br>B ( ) Office worker / Technician<br>C ( ) Middle manager (Quadro)<br>D ( ) Executive / Senior manager (Dirigente)<br>E ( ) Self-employed professional<br>F ( ) Other	
• NOT EMPLOYED	1 ( ) Seeking work<br>2 ( ) Not seeking work	

PATIENT'S FAMILY SITUATION ____________________________

CURRENT STATUS OF THE PARENTS (or legal guardians):

1 ( ) MARRIED / COHABITING   2 ( ) SEPARATED / DIVORCED   3 ( ) OTHER……………………

A ( ) Joint custody
B ( ) Sole custody

A ( ) Primary residence with mother
B ( ) Primary residence with father
C ( ) Shared residence

MOTHER

**Age:** ……………………	**Education level:**	**Occupation:**
	1 ( ) Compulsory schooling	1 ( ) Not employed / retired
	2 ( ) Vocational qualification	2 ( ) Manual worker / craftsperson
	3 ( ) Upper secondary diploma	3 ( ) Office worker / technician
	4 ( ) University degree or higher	4 ( ) Executive / manager

FATHER

**Age:** ……………………	**Education level:**	**Occupation:**
	1 ( ) Compulsory schooling	1 ( ) Not employed / retired
	2 ( ) Vocational qualification	2 ( ) Manual worker / craftsperson
	3 ( ) Upper secondary diploma	3 ( ) Office worker / technician
	4 ( ) University degree or higher	4 ( ) Executive / manager

OTHER MEMBERS OF THE IMMEDIATE HOUSEHOLD (LIVING TOGETHER)

**Member**	**Age**	**Occupation**		
( ) Brother / ( ) Sister	…………..	Student (grade: ……………………)	Employed	Other
( ) Brother / ( ) Sister	…………..	Student (grade: ……………………)	Employed	Other
( ) Brother / ( ) Sister	…………..	Student (grade: ……………………)	Employed	Other
( ) Brother / ( ) Sister	…………..	Student (grade: ……………………)	Employed	Other
Other: ………………	…………..	Student (grade: ……………………)	Employed	Other
Other: ………………	…………..	Student (grade: ……………………)	Employed	Other

GRANDPARENTS (ASCENDANT FAMILY MEMBERS)

**Family member**	**Alive**		**Education level**		
Maternal grandfather	YES	NO	1 ( ) Compulsory school	2 ( ) Upper secondary diploma	3 ( ) University
Maternal grandmother	YES	NO	1 ( ) Compulsory school	2 ( ) Upper secondary diploma	3 ( ) University
Paternal grandfather	YES	NO	1 ( ) Compulsory school	2 ( ) Upper secondary diploma	3 ( ) University
Paternal grandmother	YES	NO	1 ( ) Compulsory school	2 ( ) Upper secondary diploma	3 ( ) University

FAMILY SOCIO-ECONOMIC SITUATION

Please list the SOURCES OF INCOME for your household BEFORE the illness (you may tick more than one):

□ Salary NO YES [Number of family members receiving a salary: …………….]

□ Self-employment income NO YES [Number of self-employed family members: …………………]

□ Retirement pension NO YES [Number of retired family members: …………………………..…]

□ Parent's disability pension NO YES [Number of family members receiving: ……………………..…]

□ Income from assets (property, land, other) NO YES

Please list the SOURCES OF INCOME for your household AFTER the illness (you may tick more than one):

□ Salary NO YES [Number of family members receiving a salary: …………….]

□ Self-employment income NO YES [Number of self-employed family members: …………………]

□ Retirement pension NO YES [Number of retired family members: …………………………..…]

□ Parent's disability pension NO YES [Number of family members receiving: ……………………..…]

□ Income from assets (property, land, other) NO YES

FINANCIAL BENEFITS AND ENTITLEMENTS RELATED TO THE ILLNESS
(during and after your child's illness)

Non-self-sufficiency funds (Lombardy residents: Measures B1 & B2)	NO	YES
Law 104/1992 (disability support)	NO	YES
Disability allowance granted to your child	NO	YES
Attendance allowance *(indennità di accompagnamento)* granted to your child	NO	YES
Financial support from patient associations	NO	YES
Financial support from family members	NO	YES
Other forms of financial support (municipal grants, associations, other…)	NO	YES

PATIENT LIFE HISTORY

The following questions help reconstruct the developmental history:

Maternal chronic illnesses prior to pregnancy NO YES

Need for the mother to take medications continuously during pregnancy NO YES

Pregnancy complications (preeclampsia or eclampsia, gestational diabetes, excess or reduced amniotic fluid, etc.) NO YES

Alcohol consumption during pregnancy NO Occasional Daily

Tobacco use during pregnancy NO Occasional Daily

Exposure to diagnostic imaging during pregnancy (X-rays, CT, MRI) NO YES

Exposure to highly stressful events during pregnancy (bereavement, accidents, relocations, emotional shock, etc.) NO YES

Preterm birth (before the 38th week) NO YES

Post-term birth (after the 42nd week) NO YES

Need for induction of labor via medications (oxytocin, etc.) NO YES

Use of assisted delivery techniques (forceps, vacuum, etc.) NO YES

Cesarean delivery NO YES

APGAR score at birth (if unknown, please tick the box) ………………….. and ………………….

Maternal postpartum depression (as diagnosed by a specialist) NO YES

At approximately how many months did the child begin to walk independently? …………………..

Delay in language acquisition NO YES

Difficulties with sphincter control after age 3 (urinary and/or fecal incontinence) NO Occasional *(max 2 times per month)* Frequent *(once per week)*

Family history of developmental disorders (delay, autism) or central nervous system diseases (epilepsy, etc.) NO YES

SIGNIFICANT FAMILY EVENTS BEFORE YOUR CHILD'S ILLNESS

Other illnesses requiring hospitalization of your child	NO	YES
Illnesses requiring hospitalization of other family members	NO	YES
Bereavements (deaths in the family)	NO	YES
Relocation / move	NO	YES
Parental employment problems	NO	YES
Financial difficulties	NO	YES
Legal issues affecting family life	NO	YES
Other	NO	YES

SIGNIFICANT FAMILY EVENTS AFTER YOUR CHILD'S ILLNESS

Other illnesses requiring hospitalization of your child	NO	YES
Illnesses requiring hospitalization of other family members	NO	YES
Bereavements (deaths in the family)	NO	YES
Relocation / move	NO	YES
Parental employment problems	NO	YES
Financial difficulties	NO	YES
Legal issues affecting family life	NO	YES
Other	NO	YES

PATIENT’S SCHOOL AND EXTRACURRICULAR HISTORY

Before the illness, did your child attend school (preschool/kindergarten, primary, secondary)?
( ) NO ( ) YES

If **YES**, please describe your child’s **SCHOOL ROUTINE BEFORE THE ILLNESS**
School schedule: 1 ( ) Morning only 2 ( ) 2–3 afternoon sessions per week 3 ( ) Full-time

After-school arrangements: 1 ( ) Parent/siblings 2 ( ) Grandparents
3 ( ) Babysitter / relatives / friends 4 ( ) After-school programs / associations

During the illness, did your child attend school (preschool/kindergarten, primary, secondary)?
( ) NO ( ) YES

If **YES**, please describe your child’s **SCHOOL ROUTINE DURING THE ILLNESS**

1 ( ) **Regular attendance**
(the school schedule was compatible with hospital appointments)

2 ( ) **Occasional absences**
(the child rarely had to miss school because of hospital appointments)

3 ( ) **Partial attendance**
(the child attended in person between therapy cycles and/or with a reduced timetable)

4 ( ) **No attendance**
(school attendance was entirely or almost entirely precluded)

Please indicate how any school absences **during treatment** were compensated (you may tick more than one):
□ Hospital school □ Home instruction □ Distance learning □ Homeschooling (family instruction) □ Other

After the illness, has your child resumed attending school (preschool/kindergarten, primary, secondary)?
( ) NO ( ) YES

If **YES**, please describe your child’s **SCHOOL ROUTINE AFTER THE ILLNESS**
School schedule: 1 ( ) Morning only 2 ( ) 2–3 afternoon sessions per week 3 ( ) Full-time

After-school arrangements: 1 ( ) Parent/siblings 2 ( ) Grandparents
3 ( ) Babysitter / relatives / friends 4 ( ) After-school programs / associations

EXTRACURRICULAR LIFE BEFORE THE ILLNESS

Please list below any sports or other extracurricular/recreational activities (e.g., musical instrument, drawing/art, theatre, other) undertaken by your child:

………………………………………………………………….. For how many years? …………………
………………………………………………………………….. For how many years? …………………
………………………………………………………………….. For how many years? …………………

EXTRACURRICULAR LIFE AFTER THE ILLNESS
Please list any sports or other extracurricular/recreational activities (e.g., musical instrument, drawing/art, theatre, other) undertaken by your child:

……………………………………………………………….. For how many years? …………………
……………………………………………………………….. For how many years? …………………
……………………………………………………………….. For how many years? …………………

SCHOOL CERTIFICATIONS BEFORE THE ILLNESS
• BES *(special educational needs)* NO YES
• DSA *(specific learning disorders)* NO YES

If **YES**, this entailed the adoption of:
• **PEI (Individualized Education Plan)** NO YES
□ Support teacher — hours/week: ……………………
□ Educator — hours/week: ……………………………
• **PDP (Personalized Didactic Plan)** NO YES

SCHOOL CERTIFICATIONS AFTER THE ILLNESS
• BES *(special educational needs)* NO YES
• DSA *(specific learning disorders)* NO YES

If **YES**, this entailed the adoption of:
• **PEI (Individualized Education Plan)** NO YES
□ Support teacher — hours/week: ……………………
□ Educator — hours/week: ……………………………
• **PDP (Personalized Didactic Plan)** NO YES

Student's academic strengths BEFORE the illness:
□ Motivation and cooperation
□ Participation and integration in class
□ Attention and concentration
□ Memory skills
□ Problem-solving skills and/or insight and/or creativity
□ Autonomy / independence
□ Other: …………………………………………………………………...

Student's academic strengths AFTER the illness:
□ Motivation and cooperation
□ Participation and integration in class
□ Attention and concentration
□ Memory skills
□ Problem-solving skills and/or insight and/or creativity
□ Autonomy / independence
□ Other:
………

School performance BEFORE the illness:
1 ( ) Insufficient 2 ( ) Sufficient 3 ( ) Fair 4 ( ) Good 5 ( ) Excellent 6 ( ) Not graded

School performance AFTER the illness:
1 ( ) Insufficient 2 ( ) Sufficient 3 ( ) Fair 4 ( ) Good 5 ( ) Excellent 6 ( ) Not graded

PATIENT’S CLINICAL HISTORY

What type of **DIAGNOSIS** did your child receive?
………

Please indicate the **month and year** when your child received the initial diagnosis of the disease:
……

Please tick what your child’s **treatment protocol** included:
□ Surgery □ Chemotherapy □ Radiotherapy □ Other: …………………………… □ I don’t know

Is your child aware of their own medical history? □ YES □ NO

If **YES**, when did your child become aware of their medical history?
1 ( ) From the time of diagnosis 2 ( ) Gradually during treatment
3 ( ) After completing treatment 4 ( ) Recently

From whom did your child seek information about their illness? *(you may select more than one)*
□ Parents
□ Other relatives (siblings, grandparents, aunts/uncles)
□ Significant non-family adults (friends, neighbors, etc.)
□ Friends/peers

□ Teachers
□ Classmates
□ Healthcare professionals (physicians, nurses, healthcare assistants, therapists)
□ Other patients

Please tick the aspects of the illness about which your child has asked or sought information *(you may tick more than one)*

A. Illness
□ Frequency
□ Causes of the illness
□ Symptoms of the illness
□ Prognosis and survival
□ Outcomes and consequences of the illness
□ None of the above / has never asked questions about the illness
□ Other (please specify): ……………………………………………………………

B. Medications and therapies
□ Purpose of the medications
□ How the medications work
□ How medications are taken/ administered
□ Side effects of the medications
□ None of the above / has never asked questions about medications and therapies
□ Other (please specify): ……………………………………………………………

C. Diagnostic tests and surgery
□ Purpose of the tests or procedure(s)
□ How the tests work
□ Preparation for the test/procedure
□ None of the above / has never asked questions about tests and surgical procedures
□ Other (please specify): ……………………………………………………………

The following items concern any **problems or difficulties** your child may have after the oncological illness. Please tick to indicate their presence.

□ Aesthetic/appearance problems (alopecia, scars, other)
□ Sensory problems (vision, hearing)
□ Motor problems (due to neurological or orthopedic causes)
□ Speech/language problems
□ Growth problems
□ Learning problems

Following the illness, did your child need:

□ **Physiotherapy** [if yes, for how long? ……………………………..………]
□ **Psychomotor therapy** [if yes, for how long? ……………………………..………]
□ **Speech therapy** [if yes, for how long? ……………………………..………]
□ **Neuropsychological intervention** [if yes, for how long? ……………………………..………]
□ **Psychologist / Psychotherapist** [if yes, for how long? ……………………………..………]
□ **Educator** [if yes, for how long? ……………………………..………]
□ **Assistive devices** *(wheelchair, tripod cane, walker, magnifier, keyboard, desk, speech synthesizers, specialized software, ...)* [if yes, for how long? ……………………………..………]
□ **Other:** ……………………………… [if yes, for how long? ……………………………..………]

PSYCHOLOGICAL–BEHAVIORAL PROFILE OF THE PATIENT

Please complete the following checklist to describe your child's psychological–behavioral profile **BEFORE the illness**:

Separation anxiety problems	NO	YES
School-related anxiety problems	NO	YES
Phobias	NO	YES
Mood problems/disturbances	NO	YES
Self-esteem problems	NO	YES
Mood swings	NO	YES
Irritability	NO	YES
Behavioral problems	NO	YES
Difficulties with rules/limits	NO	YES
Low frustration tolerance	NO	YES
Limited sustained attention	NO	YES
Distractibility	NO	YES
Easy fatigability	NO	YES
Slowing *(psychomotor)*	NO	YES
Passivity	NO	YES
Relationship difficulties	NO	YES
OTHER	NO	YES

Has your child undergone a **PSYCHOLOGICAL ASSESSMENT BEFORE** the illness? □ NO □ YES
[If yes, please summarize the overall results below:
………
………
……]

Has your child undergone a **COGNITIVE ASSESSMENT BEFORE** the illness? □ NO □ YES
[If yes, please summarize the overall results below:
………
………
……]

Did your child have **difficulties integrating with peers BEFORE** the illness? □ NO □ YES

Have you ever thought your child had **few friends BEFORE** the illness? □ NO □ YES

PSYCHOLOGICAL–BEHAVIORAL PROFILE OF THE PATIENT ___________________

Please complete the following checklist to describe your child's psychological–behavioral profile **AFTER the illness and currently**:

Separation anxiety problems	NO	YES
School-related anxiety problems	NO	YES
Phobias	NO	YES
Mood problems/disturbances	NO	YES
Self-esteem problems	NO	YES
Mood swings	NO	YES
Irritability	NO	YES
Behavioral problems	NO	YES
Difficulties with rules/limits	NO	YES
Low frustration tolerance	NO	YES
Limited sustained attention	NO	YES
Distractibility	NO	YES
Easy fatigability	NO	YES
Slowing *(psychomotor)*	NO	YES
Passivity	NO	YES
Relationship difficulties	NO	YES
OTHER	NO	YES

Has your child undergone a **psychological assessment AFTER** the illness? □ NO □ YES
[If yes, please summarize the overall results below:
……
……
……………………………………………………………………………………………………]

Has your child undergone a **cognitive assessment AFTER** the illness? □ NO □ YES
[If yes, please summarize the overall results below:
……
……
……………………………………………………………………………………………………]

Has your child had **difficulties integrating with peers AFTER** the illness? □ NO □ YES

Have you ever thought your child has **few friends AFTER** the illness? □ NO □ YES
