# Supplementary material for: Psychosocial, Neuropsychological, Academic, and Social Outcomes in Pediatric Solid Tumor Survivors: An Exploratory Parent-Reported Study
Source: Children (Basel). 2026 Jul 18;13(7):943. doi: 10.3390/children13070943 (PMC13406780; doi:10.3390/children13070943)
Supplement: Supplementary file 1 [file children-13-00943-s001.zip › Supplementary Material File S2.pdf]

Operational definitions and coding rules

Within the purpose-built questionnaire, variables that were not purely descriptive were operationalized according to their specific prespecified coding rules. Checklist-style items were generally summarized as count scores, with each endorsed sub-aspect contributing one point to the relevant total. For checklist-derived difficulty scores, higher values indicate a greater reported burden; for adaptive-resource scores, higher values indicate greater reported resources. Other derived variables were coded as binary, ordinal, categorical, or continuous measures, as appropriate. Exact wording, coding rules, and permissible ranges for each item are detailed below. **Demographic variables** included age at the time of questionnaire completion (years), sex (male/female), and patient main activity (student, employed, unemployed, unspecified).

Clinical variables included age at diagnosis (years), time since diagnosis (months), detailed tumor diagnosis (bone sarcoma, CNS germ cell tumors, CNS other malignant tumors, extra-CNS germ cell tumor, ganglio/neuroblastoma, Hodgkin and non-Hodgkin lymphoma, low-grade gliomas, other), tumor site (CNS versus non-CNS tumors), and treatment modalities (surgery, chemotherapy, radiotherapy, other). Appearance- and growth-related consequences were summarized as a count ranging from 0 to 2. Learning and language-related difficulties were summarized as a count ranging from 0 to 4 and including sensory, motor, speech/language, and learning difficulties. Rehabilitation needs were summarized as physical rehabilitation needs (0-2: physiotherapy and assistive devices), neurodevelopmental rehabilitation needs (0-3: psychomotor therapy, speech/language therapy, and neuropsychological intervention), and psychological/educational intervention needs (0-2: psychologist/psychotherapist and educator). Additional clinical data included disease awareness (none, early-during treatment, late-after treatment), number of people consulted about the illness (0-8), and the number of illness-related topics the patient inquired about (0-3).

Family variables included parents' ages, marital status (cohabiting, separated, other), presence of siblings (yes/no), and number of living grandparents (0-4).

Cultural variables included the family educational level, calculated as the sum of the schooling levels of parents and grandparents (0-4 each), divided by the number of contributors; geographic area of residence (north, center, south of Italy); and municipality size (<20,000 inhabitants = small; ≥20,000 inhabitants = large).

Economic variables included parents' occupation (worker/artisan, clerk/technician, manager/executive, unemployed/retired), a weighted household income-source score based on the presence of five different source incomes (0-4.3) and categorized as low (<2), middle (=2), or high (>2), and number of financial aids or benefits received (0-7).

Premorbid characteristics included prenatal and perinatal complications (0-17); internalizing problems (anxiety, phobias, mood, and self-esteem; 0-6); externalizing problems (irritability, behavior, rule compliance, frustration tolerance; 0-4); neuropsychological difficulties (attention, distractibility, fatigue, psychomotor slowing, passivity; 0-6); and adaptive resources (0-6). Additional variables included number of extracurricular activities (free text), relational difficulties (yes/no), social integration issues (0-2, based on integration and number of friends), history of psychological or cognitive evaluations (yes/no), academic performance (excellent, good, fair, sufficient), use of personalized education plans (yes/no), and traumatic or adverse experiences (0-8), including hospitalizations or bereavement (0-3), relocation, employment, financial, or legal family issues (0-5).

When the same construct was available at both time points, change categories were derived from the within-person difference between the parent-reported post-treatment/current score and the retrospectively reported pre-diagnosis score, and were classified as improved, stable, or worsened.
