# Supplementary material for: Psychosocial, Neuropsychological, Academic, and Social Outcomes in Pediatric Solid Tumor Survivors: An Exploratory Parent-Reported Study
Source: Children (Basel). 2026 Jul 18;13(7):943. doi: 10.3390/children13070943 (PMC13406780; doi:10.3390/children13070943)
Supplement: Supplementary file 1 [file children-13-00943-s001.zip › Supplementary Tables.pdf]

Supplementary Table S1. Operational definitions of parent-reported functional outcomes and post-treatment assessment use

Outcome domain	Parent-reported indicators
**Psychological outcomes**	• Worsening of internalizing difficulties, including separation anxiety, school-related anxiety, phobias, mood problems, self-esteem problems, and mood swings.<br>• Worsening of externalizing difficulties, including irritability, behavioural problems, difficulties with rules/limits, and low frustration tolerance.<br>• Psychological assessment after cancer.
**Neuropsychological outcomes**	• Worsening of neuropsychological difficulties, including limited sustained attention, distractibility, easy fatigability, psychomotor slowing, passivity, and other reported difficulties.<br>• Reduction in adaptive resources, including motivation and cooperation, participation and integration in class, attention and concentration, memory skills, problem-solving/insight/creativity, and autonomy/independence.<br>• Cognitive assessment after cancer.
**Social outcomes**	• Worsening of relational difficulties.<br>• Worsening of social integration difficulties.<br>• Reduction in extracurricular activity participation.
**Academic outcomes**	• Worsening of academic performance.<br>• New or worsened need for an Individualized Education Plan or Personalized Didactic Plan (PEI/PDP).

For outcomes based on pre- and post-treatment comparisons, worsening was defined as a worse parent-reported post-treatment status relative to retrospectively reported pre-diagnosis functioning. For binary analyses, worsening was compared with stable or improved functioning.

All measures were derived from a purpose-built parent/caregiver questionnaire and represent proxy-reported indicators rather than clinical diagnoses or standardized neuropsychological assessments.

Supplementary Table S2. Questionnaire-derived candidate variables, operational definitions, and role in the analyses

Conceptual domain	Variables	Operational definition and role in the revised analyses
**Demographic characteristics**	Survivor age at questionnaire completion; sex	Age was recorded in years and, where modelled, expressed per interquartile range increase. Sex was coded as female versus male. Both were considered in the exploratory screen; sex was additionally included in the multivariable model for externalizing difficulties.
	Current main activity	Categorized as student, employed, unemployed, or not specified. Descriptive variable only.
**Clinical characteristics**	Age at diagnosis; time since diagnosis	Age at diagnosis was recorded in years and, where modelled, expressed per interquartile range increase. Time since diagnosis was recorded in months. Age at diagnosis was included in the exploratory screen and in the multivariable models for neuropsychological difficulties and academic worsening; time since diagnosis was descriptive only.
	Tumor diagnosis and tumor site	Detailed diagnostic categories were recorded. Tumor site was categorized as CNS versus non-CNS tumor and was used in the CNS versus non-CNS comparisons, exploratory screen, and multivariable models.
	Treatment modalities	Surgery, chemotherapy, radiotherapy, and other treatments were recorded. For descriptive analyses, treatment was summarized as other/none, surgery only, surgery plus chemotherapy, or surgery plus chemotherapy and radiotherapy. For regression analyses, multimodal treatment was coded as surgery plus chemotherapy and radiotherapy versus all other treatment patterns.
	Post-treatment consequences and rehabilitation needs	Appearance and growth-related consequences were summarized as a count from 0 to 2. Learning and language-related difficulties were summarized as a count from 0 to 4, including sensory, motor, speech/language, and learning problems. Rehabilitation needs were summarized as physical rehabilitation needs (0–2: physiotherapy and assistive devices), neurodevelopmental rehabilitation needs (0–3: psychomotor therapy, speech therapy, and neuropsychological intervention), and psychological/educational intervention needs (0–2: psychologist/psychotherapist and educator). These variables were compared between CNS and non-CNS survivors in Table 2.
	Illness awareness and information-seeking	Disease awareness was classified as absent, early/during treatment, or late/after treatment. The number of people consulted about the illness ranged from 0 to 8; the number of illness-related topic areas asked about ranged from 0 to 3. Descriptive variables only.
**Family characteristics**	Maternal and paternal age	Recorded in years. Paternal age was included in the exploratory screen as an interquartile-range-scaled variable; maternal age was descriptive only.
	Parents' marital status	Categorized as cohabiting, separated/divorced, or other. For regression analyses, parental separation was coded as yes for separated parents and no for all other reported marital-status categories.
	Siblings and grandparents	Presence of siblings was coded yes/no; number of siblings was analysed as a continuous variable, expressed per interquartile range increase in the exploratory screen. Number of living grandparents ranged from 0 to 4. Birth order and living grandparents were descriptive variables only.
**Cultural characteristics**	Family educational level	Calculated as the mean available ordinal education score across parents and grandparents. It was categorized as middle/low (<2) or middle/high (≥2). Middle/high education was included in the exploratory screen and in all multivariable models.
	Geographic residence and municipality size	Geographic residence was categorized as Northern, Central, or Southern Italy. Municipality size was classified as small (<20,000 inhabitants) or large (≥20,000 inhabitants). Both variables were included as broad place-based contextual proxies in the exploratory univariable screen. Municipality size was analysed using univariable Firth logistic regression, whereas geographic residence was assessed using a global Monte Carlo Fisher–Freeman–Halton test because of sparse geographic strata. Neither residential variable was included in the multivariable models because of the limited sample size.

**Socio-economic characteristics**	Parental occupation	Maternal and paternal occupation were categorized as unemployed/retired, worker/artisan, clerk/technician, or manager/executive. Descriptive variables only.
	Household income sources before illness	A weighted income-source score was calculated from salary, self-employment income, retirement pension, disability pension, and income from assets. Scores ranged from 0 to 4.3 and were categorized as low (<2), middle (=2), or high (>2). Descriptive variable only.
	Financial benefits related to illness	Number of financial benefits or entitlements received during or after illness, ranging from 0 to 7. Descriptive variable only.
**Premorbid developmental, psychological, and educational characteristics**	Prenatal and perinatal complications	Count score ranging from 0 to 17, based on reported prenatal, perinatal, developmental, and family-history indicators. Descriptive variable only.
	Premorbid internalizing, externalizing, and neuropsychological difficulties	Count scores based on parent-reported difficulties before diagnosis: internalizing difficulties, 0–6; externalizing difficulties, 0–4; neuropsychological difficulties, 0–6. In the exploratory screen, these were dichotomized as any difficulty versus none. Premorbid externalizing and neuropsychological difficulties were also included in their corresponding multivariable models.
	Premorbid adaptive resources	Count score from 0 to 6, including motivation/cooperation, participation/integration, attention/concentration, memory, problem solving/creativity, and autonomy/independence. Higher scores indicate greater reported adaptive resources. Descriptive variable only.
	Premorbid social, activity, and assessment indicators	Number of extracurricular activities was recorded from free-text responses. Relational difficulties were coded yes/no; social integration difficulties ranged from 0 to 2; previous psychological and cognitive assessments were coded yes/no. Descriptive variable only.
	Premorbid academic indicators	Academic performance was recorded as an ordinal category. Previous PEI/PDP use was coded yes/no and was included in the exploratory screen and multivariable academic-worsening model.
	Premorbid adverse family experiences	Count score ranging from 0 to 8, including hospitalizations, bereavements, relocation, parental employment problems, financial difficulties, legal issues, and other relevant family events. Descriptive variable only.

Abbreviations: CNS, central nervous system; PDP, Personalized Didactic Plan; PEI, Individualized Education Plan.

For repeated parent-reported constructs, post-treatment status was compared with retrospectively reported pre-diagnosis status. For difficulty counts, a higher post-treatment score indicated worsening; for adaptive resources and extracurricular activities, a lower post-treatment value indicated worsening. Psychological and cognitive assessments indicate assessment use and were not interpreted as functional outcomes.

All questionnaire-derived measures are parent/caregiver proxy-reported contextual indicators and do not constitute clinical diagnoses or standardized neuropsychological assessments. Because of the limited sample size, formal exploratory association analyses were restricted to a reduced set of clinically interpretable candidate variables; remaining variables were summarized descriptively or used in CNS versus non-CNS comparisons.

Supplementary Table S3. Completeness of raw questionnaire response fields summarized by questionnaire item or item block

Questionnaire item or item block	Underlying raw response fields, n	Applicable response cells, n	Missing response cells, n (%)	Applicable participants, n	Participants with ≥1 missing response, n (%)
**Participant information and current status**					
Questionnaire respondent	1	93	0 (0.0)	93	0 (0.0)
Survivor month and year of birth	1	93	0 (0.0)	93	0 (0.0)
Survivor sex	1	93	0 (0.0)	93	0 (0.0)
Geographic residence	1	93	3 (3.2)	93	3 (3.2)
Municipality size	1	93	3 (3.2)	93	3 (3.2)
Current main activity	1	93	1 (1.1)	93	1 (1.1)
Current educational level	1	93	12 (12.9)	93	12 (12.9)
Current school grade/course	1	93	15 (16.1)	93	15 (16.1)
**Family situation**					
Parents' marital status	1	93	0 (0.0)	93	0 (0.0)
Maternal characteristics: age, education, and occupation	3	279	4 (1.4)	93	2 (2.2)
Paternal characteristics: age, education, and occupation	3	279	10 (3.6)	93	4 (4.3)
Sibling composition: number of siblings and birth order	2	186	0 (0.0)	93	0 (0.0)
Ages of siblings living in the household	4	89	0 (0.0)	66	0 (0.0)
Grandparent living status and education	8	744	36 (4.8)	93	6 (6.5)
**Family socio-economic situation**					
Household income sources before illness	5	465	0 (0.0)	93	0 (0.0)
Household income sources after illness	5	465	0 (0.0)	93	0 (0.0)
Parental employment changes related to the illness	2	186	0 (0.0)	93	0 (0.0)
Financial benefits and entitlements related to the illness	7	651	0 (0.0)	93	0 (0.0)
**Patient life history**					
Pre-pregnancy, pregnancy, developmental, and family-history indicators	17	1581	13 (0.8)	93	1 (1.1)
Recorded APGAR score at birth	1	93	88 (94.6)	93	88 (94.6)
Health-related family events before illness	3	279	0 (0.0)	93	0 (0.0)
Socio-economic and other family events before illness	5	465	0 (0.0)	93	0 (0.0)
Health-related family events after illness	3	279	2 (0.7)	93	1 (1.1)
Socio-economic and other family events after illness	5	465	5 (1.1)	93	1 (1.1)
**School and extracurricular history**					
School attendance before illness	1	93	5 (5.4)	93	5 (5.4)
School attendance during illness	1	93	5 (5.4)	93	5 (5.4)
School routine during illness	1	93	9 (9.7)	93	9 (9.7)
Compensation of school absences during treatment	1	93	21 (22.6)	93	21 (22.6)
School attendance after illness	1	93	7 (7.5)	93	7 (7.5)
School schedule after illness	1	93	12 (12.9)	93	12 (12.9)
After-school arrangements after illness	1	93	13 (14.0)	93	13 (14.0)
Extracurricular activities before illness	1	93	6 (6.5)	93	6 (6.5)
Extracurricular activities after illness	1	93	5 (5.4)	93	5 (5.4)

School certifications before illness	1	93	4 (4.3)	93	4 (4.3)
PEI/PDP before illness	1	93	4 (4.3)	93	4 (4.3)
Academic strengths before illness	6	558	102 (18.3)	93	17 (18.3)
School performance before illness	1	93	8 (8.6)	93	8 (8.6)
School certifications after illness	1	93	4 (4.3)	93	4 (4.3)
PEI/PDP after illness	1	93	4 (4.3)	93	4 (4.3)
Academic strengths after illness	6	558	114 (20.4)	93	19 (20.4)
School performance after illness	1	93	6 (6.5)	93	6 (6.5)
**Clinical history**					
Tumor diagnosis	1	93	0 (0.0)	93	0 (0.0)
Month and year of initial diagnosis	1	93	2 (2.2)	93	2 (2.2)
Treatment protocol components	4	372	0 (0.0)	93	0 (0.0)
Awareness of own medical history	1	93	12 (12.9)	93	12 (12.9)
Timing of awareness of own medical history	1	93	3 (3.2)	93	3 (3.2)
Sources from whom the survivor sought information	8	744	9 (1.2)	93	2 (2.2)
Illness-related topics about which information was sought	3	279	3 (1.1)	93	1 (1.1)
Post-treatment appearance and growth-related difficulties	2	186	2 (1.1)	93	1 (1.1)
Post-treatment sensory, motor, speech/language, and learning difficulties	4	372	4 (1.1)	93	1 (1.1)
Post-treatment physical rehabilitation needs	2	186	2 (1.1)	93	1 (1.1)
Post-treatment neurodevelopmental rehabilitation needs	3	279	3 (1.1)	93	1 (1.1)
Post-treatment psychological and educational support needs	2	186	2 (1.1)	93	1 (1.1)
Other post-treatment support needs	1	93	1 (1.1)	93	1 (1.1)
**Psychological-behavioral profile before illness**					
Psychological-behavioral checklist before illness	17	1581	51 (3.2)	93	3 (3.2)
Psychological assessment before illness	1	93	3 (3.2)	93	3 (3.2)
Cognitive assessment before illness	1	93	3 (3.2)	93	3 (3.2)
Social integration and friendship difficulties before illness	2	186	6 (3.2)	93	3 (3.2)
**Psychological-behavioral profile after illness**					
Psychological-behavioral checklist after illness	17	1581	34 (2.2)	93	2 (2.2)
Psychological assessment after illness	1	93	2 (2.2)	93	2 (2.2)
Cognitive assessment after illness	1	93	2 (2.2)	93	2 (2.2)
Social integration and friendship difficulties after illness	2	186	4 (2.2)	93	2 (2.2)

The questionnaire comprised 60 items; selected items with separately recorded subcomponents are displayed in separate rows to permit transparent reporting of completeness. For checklist-style items, each underlying response field was assessed separately; therefore, a missing component contributes one missing response cell without classifying the full item block as entirely missing. Ages of siblings were evaluated only for recorded sibling slots supported by a nonmissing sibling count. All other blank fields were counted as missing. Blank APGAR values were counted as missing because the database did not retain a separate code for 'unknown'.

Supplementary Table S4. Detailed family structure, parental occupation, and illness-related financial support not reported in Table 1.

Characteristic	Overall, n (%)
	**N=93**
**Number of siblings**	
0	27 (29.0)
1	49 (52.7)
2	13 (14.0)
3	2 (2.2)
4	2 (2.2)
**Birth order**	
Only child	27 (29.0)
First-born	28 (30.1)
Middle-born	32 (34.4)
Last-born	6 (6.5)
**Paternal occupation**	
Manager/executive	17 (18.3)
Clerk/technician	37 (39.8)
Worker/artisan	29 (31.2)
Unemployed/retired	6 (6.5)
Not specified	4 (4.3)
**Maternal occupation**	
Manager/executive	11 (11.8)
Clerk/technician	52 (55.9)
Worker/artisan	14 (15.1)
Unemployed/retired	12 (12.9)
Not specified	4 (4.3)
**Number of types of illness-related financial support received**	
None	27 (29.0)
1-3 types	58 (62.4)
> 3 types	8 (8.6)

Birth order refers to the survivor's position among siblings. Illness-related financial support refers to the number of distinct financial benefits or entitlements reported as received during or after the cancer experience.

Supplementary Table S5. Retrospectively reported pre-diagnosis developmental, psychological, social, and educational characteristics

Characteristic	Overall, n (%)
	N=93
**Prenatal, perinatal, and early developmental risk indicators***	
0	32 (34.8)
1	25 (27.2)
≥2	35 (38.0)
**Internalizing difficulties****	
0	56 (62.2)
1	13 (14.4)
≥2	21 (23.3)
**Externalizing difficulties****	
0	66 (73.3)
1	16 (17.8)
≥2	8 (8.9)
**Neuropsychological difficulties****	
0	67 (74.4)
1	10 (11.1)
≥2	13 (14.4)
**Relational difficulties****	
No	84 (93.3)
Yes	6 (6.7)
**Social integration difficulties****	
0	75 (83.3)
1	12 (13.3)
≥2	3 (3.3)
**Adaptive resources**	
Median (first and third quartiles)	4.0 (2.0–6.0)
**Psychological assessment before diagnosis****	
No	84 (93.3)
Yes	6 (6.7)
**Cognitive assessment before diagnosis****	
No	83 (92.2)
Yes	7 (7.8)
**Academic performance before diagnosis*****	
Excellent	31 (36.5)
Good	31 (36.5)
Fair	10 (11.8)
Sufficient	2 (2.4)
Not graded	11 (12.9)
**PEI/PDP before diagnosis******	
No	79 (88.8)
Yes	10 (11.2)
**Extracurricular activities*******	
0	27 (31.0)
1	33 (37.9)
≥2	27 (31.0)
**Potentially adverse family experiences**	
0	57 (61.3)
1	25 (26.9)
≥2	11 (11.8)

Abbreviations: PDP, Personalized Didactic Plan; PEI, Individualized Education Plan.
Pre-diagnosis characteristics were reported retrospectively by the parent/caregiver at questionnaire completion and should therefore be interpreted as proxy-reported contextual indicators rather than prospectively measured baseline data. Count scores are defined in Supplementary Table 2.
*One missing value
**Three missing values
***Eight missing values
****Four missing values
*****Six missing values

Supplementary Table S6. Detailed clinical profile and post-treatment support needs not reported in Table 1.

Characteristic	Overall, n (%)
	**N=93**
**Appearance- and growth-related consequences, number of affected domains***	
0	43 (46.7)
1	41 (44.6)
2	8 (8.7)
**Learning and language-related difficulties, number of affected domains***	
0	53 (57.6)
1	26 (28.3)
2	9 (9.8)
3	3 (3.3)
4	1 (1.1)
**Physical rehabilitation needs, number of needs***	
0	59 (64.1)
1	23 (25.0)
2	10 (10.9)
**Neurodevelopmental rehabilitation needs, number of needs***	
0	72 (78.3)
1	12 (13.0)
2	5 (5.4)
3	3 (3.3)
**Psychological/educational intervention needs, number of needs***	
0	67 (72.8)
1	19 (20.7)
2	6 (6.5)

*One missing value

Appearance- and growth-related consequences include appearance-related and growth-related issues. Learning and language-related difficulties include sensory, motor, speech/language, and learning difficulties. Physical rehabilitation needs include physiotherapy and assistive devices. Neurodevelopmental rehabilitation needs include psychomotor therapy, speech/language therapy, and neuropsychological intervention. Psychological/educational intervention needs include psychologist/psychotherapist and educator support.

Supplementary Table S7. Exploratory univariable analyses of candidate variables and parent-reported outcomes.

Outcome and candidate variable	Univariable OR (95% CI)*	P value*	FDR-adjusted q value**
**Parent-reported worsening of internalizing problems**			
Outcome available: n = 90; events: n = 48			
**Sex**			
Female vs Male	2.73 (1.18-6.52)	0.018	0.344
**Tumor site**			
CNS tumor vs Non-CNS tumor	1.36 (0.59-3.17)	0.464	0.873
**Treatment**			
Surgery, chemotherapy, and radiotherapy vs All other treatment patterns	1.88 (0.77-4.76)	0.167	0.827
**Family education**			
Middle/high vs Middle/low	0.57 (0.25-1.32)	0.192	0.827
**Parental separation**			
Yes vs No	0.86 (0.24-3.13)	0.817	0.943
**Age at questionnaire completion**			
20.5 vs 10.9 years***	0.83 (0.45-1.50)	0.530	0.873
**Age at diagnosis**			
15.5 vs 6.0 years***	0.70 (0.37-1.29)	0.250	0.862
**Number of siblings**			
Per interquartile-range increase	1.20 (0.74-2.00)	0.459	0.873
**Paternal age**			
56.8 vs 46.0 years***	0.76 (0.43-1.30)	0.316	0.862
**Premorbid internalizing problems**			
Any vs None	0.81 (0.35-1.89)	0.623	0.873
**Premorbid externalizing problems**			
Any vs None	0.84 (0.33-2.11)	0.702	0.888
**Premorbid neuropsychological problems**			
Any vs None	1.88 (0.73-5.09)	0.193	0.827
**Premorbid PEI/PDP**			
Yes vs No	0.22 (0.04-0.87)	0.030	0.394
**Municipality size**			
Larger municipality/city vs small municipality	0.60 (0.22-1.57)	0.297	0.862
**Geographic area of residence********			
Global test across Northern, Central, and Southern Italy	—	0.132	0.790
**Parent-reported worsening of externalizing problems**			
Outcome available: n = 90; events: n = 29			
**Sex**			
Female vs Male	0.74 (0.30-1.77)	0.497	0.873
**Tumor site**			
CNS tumor vs Non-CNS tumor	1.75 (0.73-4.27)	0.212	0.838
**Treatment**			
Surgery, chemotherapy, and radiotherapy vs All other treatment patterns	1.27 (0.49-3.18)	0.619	0.873
**Family education**			
Middle/high vs Middle/low	0.43 (0.16-1.07)	0.071	0.605
**Parental separation**			
Yes vs No	2.31 (0.63-8.49)	0.202	0.836
**Age at questionnaire completion**			
20.5 vs 10.9 years***	0.78 (0.40-1.47)	0.447	0.873
**Age at diagnosis**			
15.5 vs 6.0 years***	0.66 (0.33-1.28)	0.223	0.838
**Number of siblings**			
Per interquartile-range increase	1.20 (0.72-1.99)	0.471	0.873
**Paternal age**			
56.8 vs 46.0 years***	0.75 (0.40-1.34)	0.336	0.873
**Premorbid internalizing problems**			
Any vs None	1.02 (0.41-2.48)	0.968	1.000
**Premorbid externalizing problems**			

Any vs None	0.65 (0.22-1.75)	0.403	0.873
**Premorbid neuropsychological problems**			
Any vs None	1.52 (0.56-3.98)	0.401	0.873
**Premorbid PEI/PDP**			
Yes vs No	1.51 (0.39-5.47)	0.536	0.873
**Municipality size**			
Larger municipality/city vs small municipality	0.83 (0.28-2.30)	0.724	0.888
**Geographic area of residence******			
Global test across Northern, Central, and Southern Italy	—	1.000	1.000
**Parent-reported worsening of neuropsychological problems**			
Outcome available: n = 90; events: n = 42			
**Sex**			
Female vs Male	1.28 (0.56-2.93)	0.557	0.873
**Tumor site**			
CNS tumor vs Non-CNS tumor	2.16 (0.94-5.09)	0.070	0.605
**Treatment**			
Surgery, chemotherapy, and radiotherapy vs All other treatment patterns	1.82 (0.75-4.49)	0.185	0.827
**Family education**			
Middle/high vs Middle/low	0.63 (0.27-1.46)	0.287	0.862
**Parental separation**			
Yes vs No	0.76 (0.20-2.72)	0.679	0.888
**Age at questionnaire completion**			
20.5 vs 10.9 years***	0.78 (0.42-1.42)	0.417	0.873
**Age at diagnosis**			
15.5 vs 6.0 years***	0.58 (0.29-1.08)	0.086	0.605
**Number of siblings**			
Per interquartile-range increase	1.77 (1.07-3.19)	0.027	0.394
**Paternal age**			
56.8 vs 46.0 years***	0.75 (0.42-1.29)	0.299	0.862
**Premorbid internalizing problems**			
Any vs None	0.85 (0.36-1.98)	0.712	0.888
**Premorbid externalizing problems**			
Any vs None	0.77 (0.30-1.93)	0.577	0.873
**Premorbid neuropsychological problems**			
Any vs None	0.85 (0.33-2.16)	0.733	0.888
**Premorbid PEI/PDP**			
Yes vs No	0.75 (0.19-2.67)	0.655	0.883
**Municipality size**			
Larger municipality/city vs small municipality	0.67 (0.24-1.76)	0.418	0.873
**Geographic area of residence******			
Global test across Northern, Central, and Southern Italy	—	0.218	0.838
**Parent-reported reduction in adaptive resources**			
Outcome available: n = 74; events: n = 22			
**Sex**			
Female vs Male	0.91 (0.33-2.42)	0.843	0.955
**Tumor site**			
CNS tumor vs Non-CNS tumor	2.88 (1.06-8.12)	0.037	0.446
**Treatment**			
Surgery, chemotherapy, and radiotherapy vs All other treatment patterns	1.30 (0.45-3.60)	0.621	0.873
**Family education**			
Middle/high vs Middle/low	0.71 (0.25-1.92)	0.504	0.873
**Parental separation**			
Yes vs No	1.28 (0.28-5.05)	0.729	0.888
**Age at questionnaire completion**			
20.5 vs 10.9 years***	0.81 (0.34-1.89)	0.626	0.873
**Age at diagnosis**			
15.5 vs 6.0 years***	1.31 (0.53-3.39)	0.561	0.873
**Number of siblings**			
Per interquartile-range increase	1.26 (0.73-2.17)	0.395	0.873
**Paternal age**			
56.8 vs 46.0 years***	0.82 (0.39-1.62)	0.577	0.873

**Premorbid internalizing problems**			
Any vs None	1.59 (0.59-4.30)	0.360	0.873
**Premorbid externalizing problems**			
Any vs None	0.95 (0.31-2.75)	0.931	0.986
**Premorbid neuropsychological problems**			
Any vs None	1.05 (0.34-3.05)	0.936	0.986
**Premorbid PEI/PDP**			
Yes vs No	1.09 (0.24-4.11)	0.904	0.977
**Municipality size**			
Larger municipality/city vs small municipality	1.23 (0.39-3.64)	0.718	0.888
**Geographic area of residence******			
Global test across Northern, Central, and Southern Italy	—	0.570	0.873
**Parent-reported worsening of relational problems**			
Outcome available: n = 90; events: n = 14			
**Sex**			
Female vs Male	1.61 (0.53-5.11)	0.400	0.873
**Tumor site**			
CNS tumor vs Non-CNS tumor	1.44 (0.47-4.46)	0.516	0.873
**Treatment**			
Surgery, chemotherapy, and radiotherapy vs All other treatment patterns	0.92 (0.25-2.93)	0.889	0.977
**Family education**			
Middle/high vs Middle/low	0.73 (0.22-2.25)	0.594	0.873
**Parental separation**			
Yes vs No	2.82 (0.61-11.18)	0.171	0.827
**Age at questionnaire completion**			
20.5 vs 10.9 years***	1.86 (0.84-4.22)	0.122	0.790
**Age at diagnosis**			
15.5 vs 6.0 years***	1.28 (0.56-2.92)	0.550	0.873
**Number of siblings**			
Per interquartile-range increase	0.74 (0.32-1.46)	0.408	0.873
**Paternal age**			
56.8 vs 46.0 years***	2.65 (1.25-6.32)	0.011	0.255
**Premorbid internalizing problems**			
Any vs None	1.30 (0.41-3.99)	0.647	0.882
**Premorbid externalizing problems**			
Any vs None	1.71 (0.50-5.40)	0.378	0.873
**Premorbid neuropsychological problems**			
Any vs None	1.26 (0.34-4.10)	0.709	0.888
**Premorbid PEI/PDP**			
Yes vs No	0.83 (0.08-4.13)	0.836	0.955
**Municipality size**			
Larger municipality/city vs small municipality	0.62 (0.11-2.36)	0.504	0.873
**Geographic area of residence******			
Global test across Northern, Central, and Southern Italy	—	1.000	1.000
**Parent-reported worsening of social integration difficulties**			
Outcome available: n = 90; events: n = 27			
**Sex**			
Female vs Male	1.34 (0.55-3.29)	0.521	0.873
**Tumor site**			
CNS tumor vs Non-CNS tumor	4.13 (1.65-10.98)	0.002	0.240
**Treatment**			
Surgery, chemotherapy, and radiotherapy vs All other treatment patterns	2.32 (0.91-5.95)	0.077	0.605
**Family education**			
Middle/high vs Middle/low	0.58 (0.22-1.45)	0.248	0.862
**Parental separation**			
Yes vs No	1.69 (0.43-6.14)	0.432	0.873
**Age at questionnaire completion**			
20.5 vs 10.9 years***	0.96 (0.49-1.82)	0.902	0.977
**Age at diagnosis**			
15.5 vs 6.0 years***	0.68 (0.33-1.32)	0.257	0.862
**Number of siblings**			

Per interquartile-range increase	0.85 (0.47-1.43)	0.554	0.873
**Paternal age**			
56.8 vs 46.0 years***	0.91 (0.49-1.63)	0.747	0.896
**Premorbid internalizing problems**			
Any vs None	1.49 (0.60-3.69)	0.391	0.873
**Premorbid externalizing problems**			
Any vs None	0.74 (0.25-2.02)	0.569	0.873
**Premorbid neuropsychological problems**			
Any vs None	1.36 (0.49-3.63)	0.541	0.873
**Premorbid PEI/PDP**			
Yes vs No	1.02 (0.23-3.76)	0.978	1.000
**Municipality size**			
Larger municipality/city vs small municipality	0.54 (0.15-1.63)	0.286	0.862
**Geographic area of residence******			
Global test across Northern, Central, and Southern Italy	—	0.864	0.969
**Worsening of academic performance**			
Outcome available: n = 85; events: n = 30			
**Sex**			
Female vs Male	0.98 (0.40-2.36)	0.961	1.000
**Tumor site**			
CNS tumor vs Non-CNS tumor	2.31 (0.94-5.75)	0.067	0.605
**Treatment**			
Surgery, chemotherapy, and radiotherapy vs All other treatment patterns	3.48 (1.37-9.14)	0.009	0.255
**Family education**			
Middle/high vs Middle/low	0.45 (0.17-1.11)	0.084	0.605
**Parental separation**			
Yes vs No	1.10 (0.29-3.79)	0.885	0.977
**Age at questionnaire completion**			
20.5 vs 10.9 years***	0.34 (0.15-0.74)	0.006	0.240
**Age at diagnosis**			
15.5 vs 6.0 years***	0.32 (0.13-0.71)	0.005	0.240
**Number of siblings**			
Per interquartile-range increase	1.39 (0.85-2.36)	0.189	0.827
**Paternal age**			
56.8 vs 46.0 years***	0.62 (0.31-1.14)	0.129	0.790
**Premorbid internalizing problems**			
Any vs None	0.90 (0.35-2.22)	0.817	0.943
**Premorbid externalizing problems**			
Any vs None	0.59 (0.20-1.62)	0.314	0.862
**Premorbid neuropsychological problems**			
Any vs None	1.58 (0.57-4.27)	0.373	0.873
**Premorbid PEI/PDP**			
Yes vs No	0.82 (0.19-3.03)	0.776	0.913
**Municipality size**			
Larger municipality/city vs small municipality	0.75 (0.25-2.10)	0.596	0.873
**Geographic area of residence******			
Global test across Northern, Central, and Southern Italy	—	0.293	0.862
**New/worsened need for PEI/PDP**			
Outcome available: n = 89; events: n = 23			
**Sex**			
Female vs Male	1.63 (0.64-4.26)	0.304	0.862
**Tumor site**			
CNS tumor vs Non-CNS tumor	1.60 (0.62-4.12)	0.332	0.873
**Treatment**			
Surgery, chemotherapy, and radiotherapy vs All other treatment patterns	2.04 (0.77-5.39)	0.152	0.827
**Family education**			
Middle/high vs Middle/low	0.96 (0.37-2.46)	0.936	0.986
**Parental separation**			
Yes vs No	0.70 (0.13-2.75)	0.633	0.873
**Age at questionnaire completion**			
20.5 vs 10.9 years***	1.13 (0.53-2.38)	0.754	0.896

**Age at diagnosis**			
15.5 vs 6.0 years***	0.65 (0.29-1.40)	0.268	0.862
**Number of siblings**			
Per interquartile-range increase	1.10 (0.63-1.85)	0.727	0.888
**Paternal age**			
56.8 vs 46.0 years***	1.16 (0.62-2.18)	0.630	0.873
**Premorbid internalizing problems**			
Any vs None	3.15 (1.20-8.64)	0.020	0.344
**Premorbid externalizing problems**			
Any vs None	1.01 (0.33-2.83)	0.983	1.000
**Premorbid neuropsychological problems**			
Any vs None	2.08 (0.73-5.81)	0.167	0.827
**Premorbid PEI/PDP**			
Yes vs No	0.11 (0.00-0.96)	0.044	0.480
**Municipality size**			
Larger municipality/city vs small municipality	0.71 (0.20-2.17)	0.564	0.873
**Geographic area of residence****			
Global test across Northern, Central, and Southern Italy	—	0.679	0.888

Abbreviations: CI, confidence interval; CNS, central nervous system; FDR, false discovery rate; OR, odds ratio; PDP, Personalized Didactic Plan; PEI, Individualized Education Plan.

*Univariable odds ratios, 95% confidence intervals, and P values were estimated using separate Firth penalized logistic regression models, except for geographic area of residence. Functional outcomes were coded as parent-reported worsening versus stable or improved functioning, relative to retrospectively reported pre-diagnosis functioning. For PEI/PDP, the event was a new or worsened need after cancer.

**Benjamini–Hochberg adjustment across all 120 screened candidate variable–outcome associations. No association remained statistically significant after FDR adjustment.

***Values represent first and third quartile of the variable distribution.

****Geographic area of residence was evaluated using a global Monte Carlo Fisher–Freeman–Halton test across Northern, Central, and Southern Italy. Category-specific odds ratios were not reported because of sparse geographic strata.

The number of observations varied slightly across individual models because of missing values in the candidate variable; it ranged from 71 to 90.
